# Effects of treatment with liraglutide on oxidative stress and cardiac natriuretic peptide levels in patients with type 2 diabetes mellitus

**DOI:** 10.1007/s12020-014-0246-6

**Published:** 2014-04-03

**Authors:** Kenta Okada, Kazuhiko Kotani, Hiroaki Yagyu, Akihiko Ando, Jun-ichi Osuga, Shun Ishibashi

**Affiliations:** 1 Division of Endocrinology and Metabolism, Department of Internal Medicine, Jichi Medical University, 3311-1 Yakushiji, Shimotsuke, Tochigi 329-0498 Japan; 2 Department of Public Health, Jichi Medical University, 3311-1 Yakushiji, Shimotsuke, Tochigi 329-0498 Japan; 3 Department of Internal Medicine, University of Tsukuba Institute of Clinical Medicine, 3-2-7 Miya-machi, Mito, Ibaraki 310-0015 Japan

## Introduction

Diabetes mellitus (DM) is a risk factor for cardiovascular disease (CVD) [1]. Both traditional and nontraditional molecules have been explored to explain the development of DM-related CVD. Molecules associated with the circulatory system, such as the natriuretic peptides, which are counterregulatory hormones involved in the regulation of volume homeostasis and cardiovascular remodeling, are an important clue for understanding the underlying mechanisms of DM-related CVD [2]. The plasma brain natriuretic peptide (BNP) level is thus a predictor of CVD events and death [3]. Additionally, the oxidative stress burden may in part explain the link between DM and CVD [4]. Although several markers are applicable, the diacron reactive oxygen metabolites (d-ROMs) test and malondialdehyde (MDA) are indicators of the global oxidative stress burden in a clinical setting [5], especially in patients with type 2 DM (T2DM) [6, 7].

Liraglutide, a long-acting glucagon-like receptor peptide (GLP)-1 analogue, is a recent treatment modality that may tolerably improve glycemic control [8–10] and provide beneficial cardiovascular effects via extrapancreatic mechanisms [11, 12]. Clinical studies of the association between liraglutide and cardiovascular risk markers, especially nontraditional markers, have been limited. The aim of the present study was to investigate the effects of 24 weeks of treatment with liraglutide on cardiovascular risk markers such as BNP, d-ROMs, and MDA in patients with T2DM.

## Subjects and methods

Sixty-five adult outpatients with T2DM (37 men and 28 women) were treated with liraglutide for 24 weeks. Eligibility criteria included the absence of pregnancy, an estimated duration of DM of no more than 15 years, no history of treatment with insulin, and a target hemoglobin A_1c_ (HbA_1c_) >7.0 % even with diet and exercise therapy. We excluded patients with a recent acute illness, inflammatory bowel disease, a history of ileus, severe nephropathy (i.e., stage 3–5), liver dysfunction, and type 1 DM. The study protocol was approved by the Jichi Medical University Ethical Committee, and written informed consent was obtained from all participants. If patients were being treated with antiglycemic agents, we discontinued use of α-glucosidase inhibitors, pioglitazone, metformin, and dipeptidyl peptidase 4 inhibitors when treatment with liraglutide was initiated because the health insurance system in Japan indicates that these medications should not be administered with liraglutide. Therefore, no hypoglycemic agents except sulfonylureas were used in this study. Treatment with liraglutide was initiated at a dosage of 0.3 mg/day and titrated up to 0.6–0.9 mg/day when necessary. The dosage of liraglutide or a sulfonylurea was decreased if the glycemic target of fasting glucose was less than 95 mg/dL or causing hypoglycemic symptoms. When treatment with liraglutide was initiated, the dosage of the sulfonylurea was decreased by half.

At baseline and after 24 weeks of treatment, weight, systolic/diastolic blood pressure, and heart rate were measured and blood/urine samples were collected for measurement of the following parameters: HbA_1c_, glucose, total cholesterol, triglyceride, high-density lipoprotein cholesterol, aspartate aminotransferase, alanine aminotransferase, γ-glutamyl transpeptidase, urine albumin, BNP, d-ROMs, and MDA. Plasma BNP levels were measured using an immunoenzymometric assay kit (E-Test TOSOH II [BNP]; Tosoh Corp., Tokyo, Japan) [13]. The d-ROMs values were obtained using a kinetic spectrophotometric assay (F.R.E.E. System; Diacron, Grosseto, Italy) [5]. The MDA values were measured using a commercially available kit (Cell Biolabs Inc., San Diego, CA, USA).

The data are presented as mean ± standard deviation for parametric variables, medians and interquartile ranges for nonparametric variables, or numbers for categorical variables. In all analyses, the variables with nonparametric distributions were treated after a log transformation. Paired *t* tests were used to examine the changes in respective parameters. Correlations between changes in variables were examined using Pearson correlation tests. Statistical significance was set at a *P* value of <0.05.

## Results

No patients dropped out of this study. The patients were 58.7 ± 10.2 years of age, body mass index was 27.9 ± 5.9 kg/m^2^, and duration of DM was 7.0 years. Before this study, 24 patients (36.9 %) had not been treated with an antidiabetic agent and 41 patients (63.1 %) had been treated with a sulfonylurea (*n* = 32), dipeptidyl peptidase 4 inhibitor (*n* = 18), or metformin (*n* = 15).

After 24 weeks of treatment with liraglutide (mean dose, 0.74 mg), both HbA_1c_ and blood glucose levels were significantly reduced (Table 1), as well as systolic and diastolic blood pressure. A significant decrease in urine albumin (mean 23.5–12.7 mg/g Cr), BNP (mean 9.2–6.3 pg/mL), and d-ROMs (mean 416.2–384.8 U. CARR) levels was also observed after treatment. There were no significant differences in body weight, body mass index, heart rate, lipid levels, liver function markers, and MDA levels.

**Table 1:** Clinical data for each variable at baseline and after treatment with liraglutide

Parameters	Baseline	Week 24	*P* value
Body weight (kg)	72.6 ± 16.0	72.1 ± 16.4	0.88
Body mass index (kg/m^2^)	27.9 ± 5.9	27.8 ± 6.0	0.93
Systolic blood pressure (mmHg)	133 ± 10	126 ± 10	<0.01**
Diastolic blood pressure (mmHg)	80 ± 10	76 ± 9	<0.02*
Heart rate (beats/min)	82 ± 13	83 ± 11	0.65
HbA1c (%)	9.6 ± 1.7	7.1 ± 0.9	<0.01**
Glucose (mg/dL)	228 ± 99	141 ± 46	<0.01**
Total cholesterol (mg/dL)	199 ± 37	189 ± 30	0.11
Triglyceride (mg/dL)	133 (92–194)	130 (94–163)	0.88
HDL cholesterol (mg/dL)	56 ± 14	56 ± 14	0.52
LDL cholesterol (mg/dL)	109 ± 35	103 ± 30	0.23
AST (IU/L)	23 (21–45)	27 (19–36)	0.71
ALT (IU/L	30 (21–45)	30 (23–42)	0.18
γ-GT (IU/L)	37 (23–57)	40 (26–57)	0.99
Urine albumin (mg/g Cr)	23.5 (11.4–46.7)	12.7 (7.2–22.4)	0.04*
BNP (pg/mL)	9.2 (5.3–16.9)	6.3 (4.1–10.9)	0.01*
d-ROMs (U. CARR)	416.2 ± 73.9	384.8 ± 64.9	0.01*
MDA (pmol/mg)	72.4 ± 36.4	69.4 ± 36.7	0.65

*HbA*_*1c*_ hemoglobin A_1c_, *HDL* high-density lipoprotein, *LDL* low-density lipoprotein, *AST* aspartate aminotransferase, *ALT* alanine aminotransferase, *GT* glutamyl transpeptidase, *BNP* brain natriuretic peptide, *d-ROMs* diacron reactive oxygen metabolites, *MDA* malondialdehyde. Data are presented as the mean ± standard deviation or median (interquartile range). *P* value comparison of respective data at baseline and after 24 weeks of treatment. Significance level: * *P* < 0.05; ** *P* < 0.01

A correlation analysis revealed that the change in BNP levels was significantly and negatively correlated to that of HbA_1c_ (*r* = − 0.30, *P* = 0.02) or positively to that of systolic blood pressure (*r* = 0.26, *P* = 0.046). The change in d-ROMs levels was significantly and positively correlated to that of MDA (*r* = 0.38, *P* < 0.01) or that of HbA_1c_ (*r* = 0.25, *P* = 0.049). The change in MDA levels was not correlated to that of HbA_1c_. The change in urine albumin levels was significantly and positively correlated to that of HbA_1c_ (*r* = 0.45, *P* < 0.01) but did not significantly correlate to the changes in blood pressure.

## Discussion

The cardioprotective effects of liraglutide, which is now widely accepted as useful for glycemic control, were described in a recent review of the literature [10]; however, these effects were examined in basic studies or short-term human trials [14], and new nontraditional markers (e.g., adipokines) have been reported to be involved in such cardioprotective mechanisms [9]. In the current study, after 24 weeks of treatment with liraglutide, a significant reduction was observed not only in levels of glucose, HbA_1c_, blood pressure, and urine albumin but also BNP and d-ROMs, which are nontraditional but potential markers that may in part explain the link between DM and CVD in patients with T2DM.

Discrepant findings of increased BNP levels resulting in weight loss and adipocyte lipolysis been reported in obese patients with T2DM [15]. The investigators discussed the effects of weight reduction on the change in BNP levels in their study [15]; the fact that no weight change was observed in the current study may partly affect this discrepancy in the direction of the change in BNP levels and merits further investigation.

The significant correlation between the change in d-ROMs levels and glycemic control in the current study might suggest a pleiotropic effect of liraglutide against CVD by alleviation of the oxidative stress burden in T2DM. The d-ROMs (reflecting the hydroperoxides of wide organic compounds of not only lipids but also proteins and nucleic acids [5]) and MDA (reflecting an end product of lipid peroxidation [7, 16]) can be different oxidative stress markers. The reduction of d-ROMs but not MDA levels indicates a possible effect of liraglutide on oxidative stress through reduced levels of proteins and/or nucleic acids more than lipid peroxidation. On the other hand, the reduction of the d-ROMs levels was not very large, so its relevance in CVD outcomes remains to be determined. A pleiotropic effect is plausible but should be further established. Additionally, this study has the limitations of a small sample size, a single-arm design, and a short-term duration. These limitations must be addressed.

In summary, treatment with liraglutide may exert a cardioprotective action and partly alleviate the burden of oxidative stress as a possible pleiotropic action in outpatients with T2DM. These data are preliminary, and future work is warranted.

## References

[1] Davidson MH (2012). Cardiovascular risk factors in a patient with diabetes mellitus and coronary artery disease: therapeutic approaches to improve outcomes: perspectives of a preventive cardiologist. Am. J. Cardiol..

[2] Levin ER, Gardner DG, Samson WK (1998). Natriuretic peptides. N. Engl. J. Med..

[3] Wang TJ, Larson MG, Levy D, Benjamin EJ, Leip EP, Omland T, Wolf PA, Vasan RS (2004). Plasma natriuretic peptide levels and the risk of cardiovascular events and death. N. Engl. J. Med..

[4] Maritim AC, Sanders RA, Watkins JB (2003). Diabetes, oxidative stress, and antioxidants: a review. J. Biochem. Mol. Toxicol..

[5] Vassalle C (2008). An easy and reliable automated method to estimate oxidative stress in the clinical setting. Methods Mol. Biol..

[6] Migdalis IN, Triantafilou P, Petridou E, Varvarigos N, Totolos V, Rigopoulos A (2005). Lipid peroxides in type 2 diabetic patients with neuropathy. Res. Commun. Mol. Pathol. Pharmacol..

[7] Sayın R, Aslan M, Kucukoglu ME, Luleci A, Atmaca M, Esen R, Demir H (2013). Serum prolidase enzyme activity and oxidative stress levels in patients with diabetic neuropathy. Endocrine.

[8] Degn KB, Juhl CB, Sturis J, Jakobsen G, Brock B, Chandramouli V, Rungby J, Landau BR, Schmitz O (2004). One week’s treatment with the long-acting glucagon-like peptide 1 derivative liraglutide (NN2211) markedly improves 24-h glycemia and alpha- and beta-cell function and reduces endogenous glucose release in patients with type 2 diabetes. Diabetes.

[9] Díaz-Soto G, de Luis DA, Conde-Vicente R, Izaola-Jauregui O, Ramos C, Romero E (2014). Beneficial effects of liraglutide on adipocytokines, insulin sensitivity parameters and cardiovascular risk biomarkers in patients with type 2 diabetes: a prospective study. Diabetes Res. Clin. Pract..

[10] C.B. Giorda, E. Nada, B. Tartaglino, Pharmacokinetics, safety, and efficacy of DPP-4 inhibitors and GLP-1 receptor agonists in patients with type 2 diabetes mellitus and renal or hepatic impairment. A systematic review of the literature. Endocrine (2014). doi:10.1007/s12020-014-0179-0 10.1007/s12020-014-0179-0 24510630

[11] Noyan-Ashraf MH, Momen MA, Ban K, Sadi AM, Zhou YQ, Riazi AM, Baggio LL, Henkelman RM, Husain M, Drucker DJ (2009). GLP-1R agonist liraglutide activates cytoprotective pathways and improves outcomes after experimental myocardial infarction in mice. Diabetes.

[12] Courrèges JP, Vilsbøll T, Zdravkovic M, Le-Thi T, Krarup T, Schmitz O, Verhoeven R, Bugáñová I, Madsbad S (2008). Beneficial effects of once-daily liraglutide, a human glucagon-like peptide-1 analogue, on cardiovascular risk biomarkers in patients with type 2 diabetes. Diabet. Med..

[13] Notsu Y, Fukuma E, Shibata H, Masuda J, Ishibashi Y (2004). Evaluation of plasma brain natriuretic peptide concentration used by AIA-360. Jpn. J. Med. Pharm. Sci..

[14] N. Mikhail, Effects of incretin-based therapy in patients with heart failure and myocardial infarction. Endocrine (2014). doi:10.1007/s12020-014-0175-4 10.1007/s12020-014-0175-4 24493030

[15] Li CJ, Yu Q, Yu P, Yu TL, Zhang QM, Lu S, Yu DM (2014). Changes in liraglutide-induced body composition are related to modifications in plasma cardiac natriuretic peptides levels in obese type 2 diabetic patients. Cardiovasc. Diabetol..

[16] Noberaseo G, Odetti P, Boeri D, Maiello M, Adezati L (1991). Malondialdehyde (MDA) level in diabetic subjects. Relationship with blood glucose and glycated hemoglobin. Biomed. Pharmacother..

